# Intradural extramedullary spinal cord meningioma with a rare extradural foraminal extension: A case report

**DOI:** 10.3389/fsurg.2023.1077355

**Published:** 2023-04-17

**Authors:** Faisal Almatrafi, Majed Alomair, Abdulrazaq Alojan, Mohammed Alkhaldi, Noor Alsafwani, Abdullah Aseeri, Abdulelah Alshahrani, Sultan Alsalmi, Mohammad Alqahtani

**Affiliations:** ^1^ Department of Neurosurgery, Imam Abdulrahman Bin Faisal University Hospital, Dammam, Saudi Arabia; ^2^ King Fahd University Hospital, Imam Abdulrahman Bin Faisal University, Al Khobar, Saudi Arabia; ^3^ College of Medicine, Imam Abdulrahman Bin Faisal University, Dammam, Saudi Arabia; ^4^ Department of Pathology, College of Medicine, Imam Abdulrahman Bin Faisal University, Dammam, Saudi Arabia

**Keywords:** meningioma, extradural, spinal, case report, intradural, extramedullary, tumor

## Abstract

**Background:**

Meningiomas are mostly benign and slow-growing neoplasms of the central nervous system. Spinal meningiomas account for up to 45% of all intradural spinal tumors in adults and up to 25%–45% of all spinal tumors. Spinal extradural meningiomas are rare and may be easily confused with malignant neoplasms.

**Case description:**

A 24-year-old woman was presented to our hospital with paraplegia and loss of sensation in the T7 dermatome and lower body. MRI findings showed T6-T7 right-sided intradural extramedullary and extradural lesion, measuring 1.4 cm × 1.5 cm × 3 cm, extending to the right foramen, compressing the spinal cord, and displacing it to the left. Hyperintense lesion on T2 and hypointense lesion on T1 were observed. The patient reported improvement after surgery and during follow-up. We recommend maximizing the decompression during surgery to achieve better clinical outcome. Extradural meningiomas represent 5% of all meningiomas; therefore, having an intradural on top of extradural meningioma with extraforaminal extensions makes this a unique and rare case.

**Conclusion:**

Meningiomas can be easily missed in diagnosis depending on imaging and the pathognomonic pattern it represents, which can mimic other pathologies, such as schwannomas. Therefore, surgeons should always suspect their patient having a meningioma even if the pattern is not typical. Moreover, preoperative preparation, such as navigation and defect closure, must be taken in case it turns out be a meningioma instead of the presumed pathology.

## 1. Introduction

Meningiomas are one of the most common primary central nervous system tumors, and they are mostly slow-growing benign tumors. Meningioma usually arises from the meningothelial cells of the arachnoid layer of the meninges. Most of these tumors (69%–79%) are World Health Organization (WHO) grade I, followed by grade II, which accounts for 20%, and grade III, with a 1%–6% percentage rate. In comparison to grade I, grades II and III harbor more aggressive behavior and rapid tumor growth (1).

Meningiomas account for approximately 45% of all the intradural tumors of the adult spine and up to 25% of all spine tumors (2). Spinal meningioma tends to originate mostly at the thoracic level. However, it can arise in other spinal levels. In most cases, spinal meningiomas are limited to the intradural space, and extradural meningiomas are rare and may be easily confused with malignant neoplasms (1). Extradural spinal meningiomas are infrequent and account for approximately 5% of all spinal meningiomas. However, the percentage of intradural extramedullary meningioma with extradural extension is not well known.

Spinal meningioma has a higher female predilection due to the higher number of estrogen receptors (ERs). Moreover, spinal meningioma tends to be WHO grade I in females, whereas males are more commonly affected with grade II and III tumors (3).

### 1.1. Meningioma in pregnancy

The biological behavior of meningioma in pregnancy is different from other meningiomas. The possible explanation is rooted in the complex physiological changes and hormonal differences during pregnancy. The increased meningioma growth observed in pregnancy is presumably the result of endocrine mechanisms. These include an increase in progesterone, estrogen, and prolactin serum levels. In contrast, the levels of pituitary hormones produced by the placenta, such as Follicle-stimulating hormone (FSH), Luteinizing hormone (LH), and human chorionic gonadotropin (hCG), decrease in the mother before childbirth. Vascular factors may also play a crucial role. Peritumoral brain edema (PTBE), with a well-known causative association with vascular endothelial growth factor (VEGF), can often be seen both with imaging and surgical specimens (4).

Meningiomas rarely develop over the course of pregnancy. There are approximately 5–6 cases out of 100,000 pregnancies. One of the reasons for this low incidence is the fact that the range of fertility is approximately 15–45 years of age, in which tumors are relatively low in frequency (5). A case report by Cushing and Eisenhardt on a pregnant woman with parasellar meningioma was issued in 1929. They reported the rapid progression of visual impairment, which was reversible postpartum and recurred in the next pregnancy (6). The background of the complex pathophysiological and morphological changes in tumors during pregnancy remains unelucidated.

In the review of the literature, there are two cardinal mechanisms to be considered, endocrine and vascular theories.

### 1.2. Endocrine mechanism

Meningioma growth is enhanced in the progesterone-dominated luteal phase of the menstrual cycle (7). During the first week of conception, estrogen and progesterone production is limited to the ovary. From the 10th week onward, the production of estrogen and progesterone is controlled by the placenta. FSH and LH levels are low due to negative feedback to the pituitary gland. Boyle-Walsh et al. demonstrated that the glycoproteins, FSH, LH, and hCG, in the *in vitro* cell culture inhibit tumor cell proliferation, whereas the proteins human placental lactogen (hPL) and prolactin (PRL) stimulate tumor propagation (8).

However, the pathognomonic effect of estrogen is questionable. ER is not expressed in most meningiomas, and this phenomenon is not different in “gestational meningiomas” (9).

The exact role of progesterone remains unelucidated despite the numerous published research papers on this topic. Most meningiomas express progesterone receptor (PR), which can also be detected by immunohistochemistry (10). The fact that tumor growth occurs in the luteal phase of the menstrual cycle or in the second or third trimester of pregnancy when progesterone plasma concentration is higher suggests the role of sex hormones in the mechanism (11).

Moreover, long-term hormone replacement therapy also raises the possibility of disease, although such a correlation has not been shown after the use of oral contraceptives (12).

### 1.3. Possible vascular mechanisms

During pregnancy, the body goes into multiple physiological changes. The dynamic circulation of the pregnant female significantly changes. Stroke volume increases up to 30%–40%. Also, the heart rate accelerates by 10–15 beats/min compared to nonpregnant females.

However, the exact role of VEGF in the growth of gestational meningioma is not clearly understood.

The body of literature suggesting the crucial role of rapid vascular changes in the mechanism is derived mainly from the morphological examination of the tumor. Some researchers have identified typical foamy, swollen, edematous cells in the histopathological meningioma samples resected from pregnant women (13). Meanwhile, other studies have found that the presence of increased vascularity and focal pathological alterations, such as intra- or extracellular edema, are significantly higher in the cases of “gestational meningioma” compared to meningiomas of nonpregnant women (9).

These tumors can be found as an incidental finding or when the tumor has grown enough to cause compressive symptoms. Clinically, patients often complain of pain at the beginning, as well as other neurological symptoms, depending on the location and the progression of compression. The diagnosis of spinal meningioma is usually delayed due to the late appearance of symptoms. The management of spinal meningioma is gross total resection, and it has a good prognosis in most cases, with results of surgical excision reported to be good to excellent with recurrence rates of 3%–7%.

Herein, we report a case of surgically resected intradural extramedullary spinal cord meningioma with extradural foraminal extension.

As mentioned, extradural meningiomas represent 5% of all meningiomas, so having an intradural on top of extradural meningioma with extraforaminal extensions makes this a unique and rare case.

## 2. Case description

A 23-year-old woman, without any history of medical illness until her seventh month of gestation, complained of right lower limb numbness and weakness, which was treated conservatively. One-week postpartum of normal vaginal delivery, the patient started to suffer from progressive bilateral lower limb weakness associated with numbness. Furthermore, the patient reported an on/off electrical sensation going down both her lower limbs. The patient sought medical attention: she was managed conservatively with analgesia, and she underwent a 13-week physiotherapy course. Despite the analgesia and physiotherapy, the patient's weakness started to worsen gradually. In addition, the patient totally lost control of her sphincters 1 month prior to the hospital presentation. The patient was investigated in another hospital and was found to have a spinal tumor in the thoracic region.

A neurological assessment revealed a bedbound patient with bilateral lower limb sensation loss associated with the bilateral loss of power, except for flickering movement in the toes. Furthermore, there was an exaggerated knee flex bilaterally, myoclonus, and hypertonia. The anal tone was absent, with an associated decrease in perianal anal sensation.

### 2.1. Preoperative imaging

**Figure 1:**
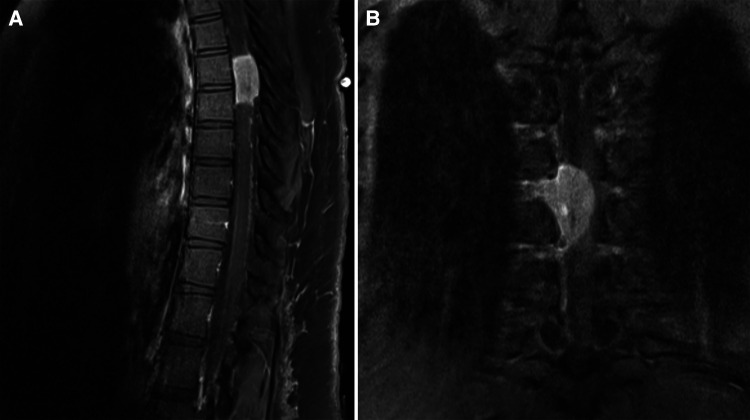
(**A**) T1W + contrast MRI sagittal view in a well-defined intradural extramedullary lesion, opposite to the T6/T7 level. (**B**) Hypointense lesion on coronal MRI T1W and enhancing lesion on T1W + contrast, with extension into the right intervertebral foramina, causing spinal cord compression, which exerts a mass effect upon the spinal cord, displacing it to the left.

**Figure 2:**
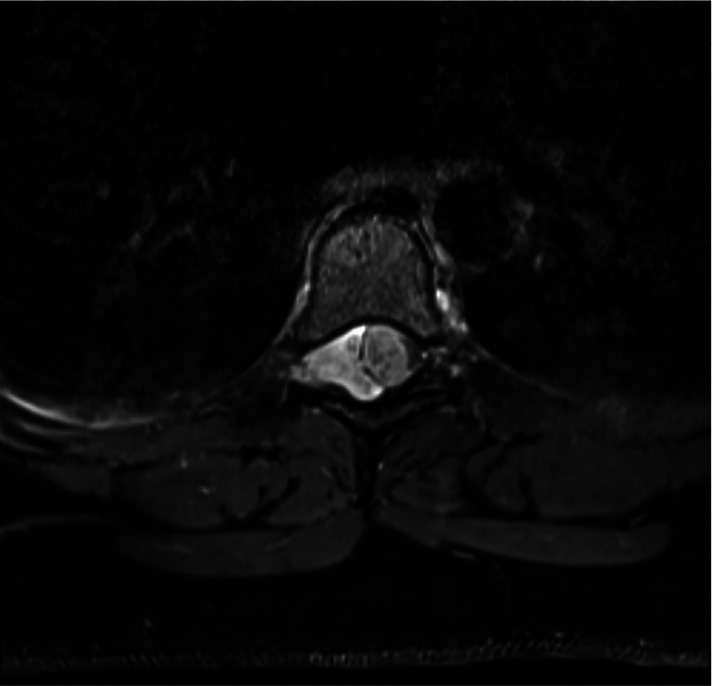
Axial MRI shows avid enhancement attachment with dural attachment, measuring 1.4 cm × 1.5 cm × 3 cm in maximum AP, TS, and CC dimensions, respectively. The spinal cord is displaced to the left.

The spine MRI on sagittal view exhibited a right-sided T6-T7 well-defined intradural extramedullary lesion, which was hyperdense on T2W (Figure 1A). On coronal view, right intervertebral foramina extension causing spinal cord compression and left cord displacement was found (Figure 1B). Axial spine MRI displayed avid enhancement with a dural attachment measuring 1.4 cm × 1.5 cm × 3 cm in maximum anterior-posterior (AP), transection (TS), and craniocaudal (CC) dimensions, respectively (Figure 2).

### 2.2. Intraoperative finding

A midline incision was performed at the T5–T7 levels, and the subcutaneous layer was dissected. The spinous process was identified, and the paraspinal muscles were pealed-off to expose the facet more on the right side. Homeostasis was maintained.

**Figure 3:**
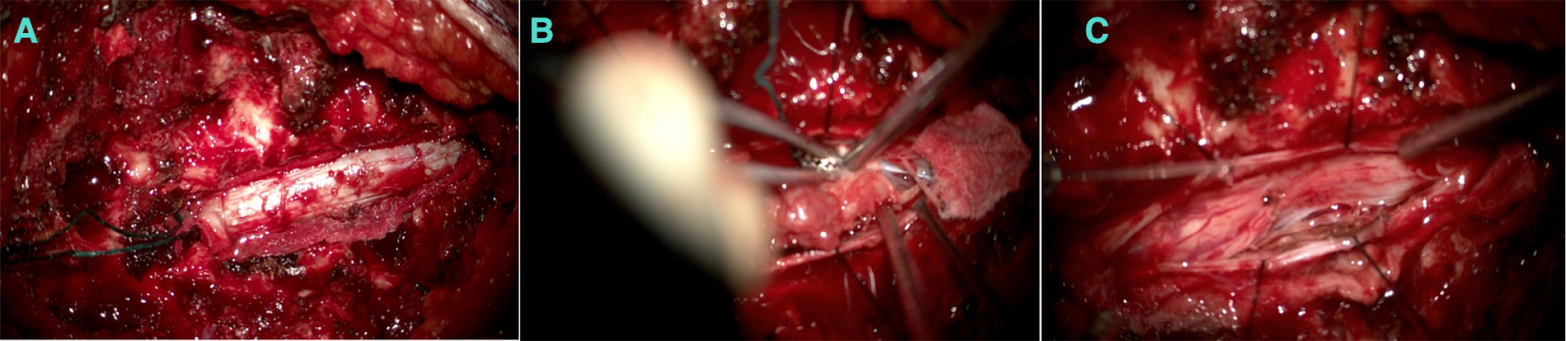
(**A**) An extradural component appearing before the dura opening. (**B**) Debulking after the intradural component. (**C**) After debulking.

Furthermore, a laminectomy of T6 and T7 levels was performed, and epidural fat and ligamentum flavum were removed (Figures 3A,B).

The extradural lesion and the foraminal part were identified and resected (Figure 3C). The dura was opened, a gush of cerebro-spinal-fluid (CSF) was observed, and the cord was pushed to the left side. The intradural component was resected carefully away from the cord, and the spinal cord was relaxed. Dura was repaired with Tacoseal and was closed in a watertight closure. Another layer of Tacoseal was applied. Tisseel was then applied. In the end, the Valsalva maneuver was performed, and there was no evidence of leak. The tumor sample was sent for histopathology.

Informed written consent was obtained from the patient.

### 2.3. Postoperative findings

#### 2.3.1. Histopathology

**Figure 4:**
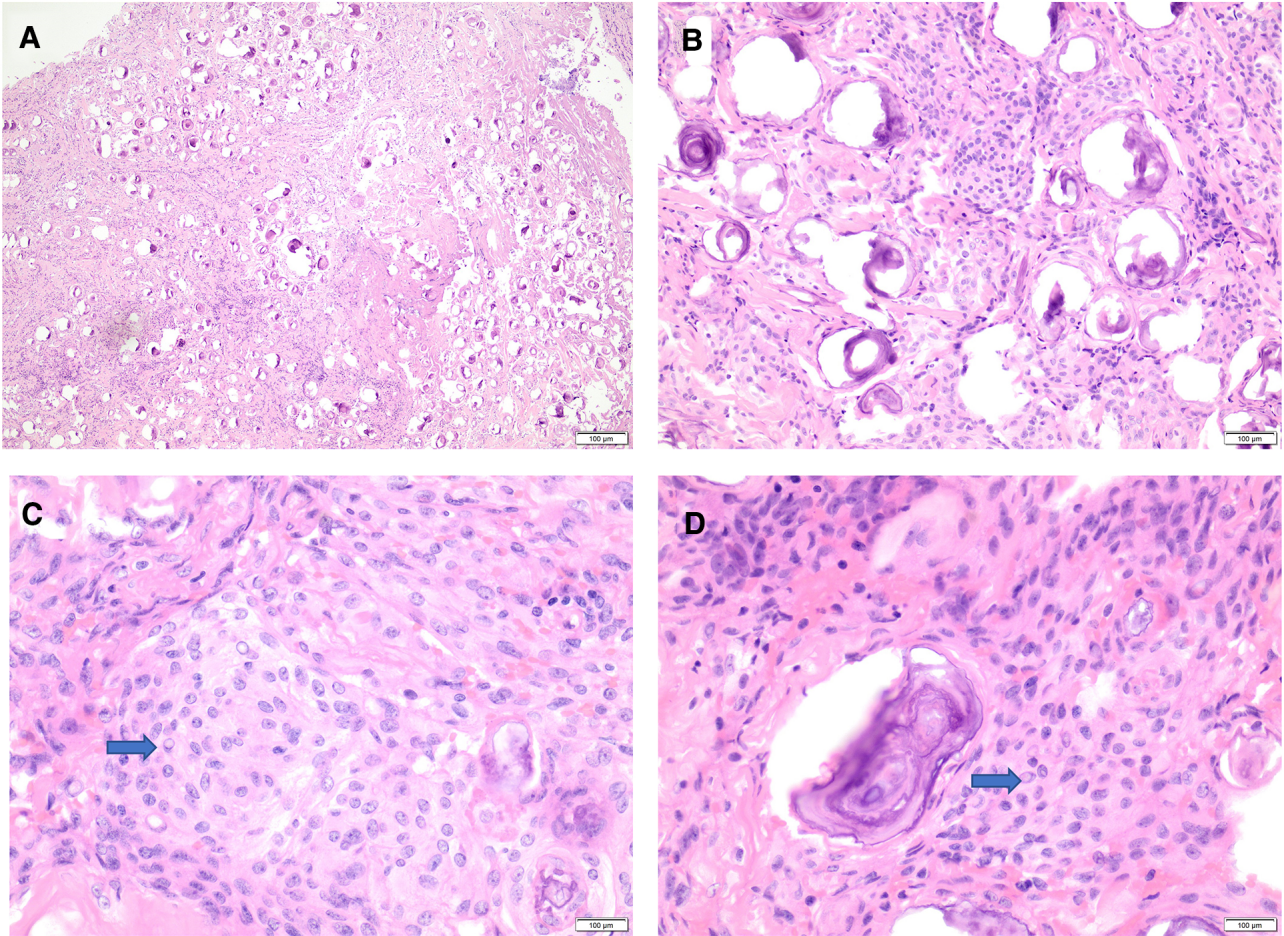
Histomorphology of psammomatous meningioma. (**A,B**) H&E stain showing meningothelial neoplasm with prominent psammomatous calcifications (**A**: 20×, B: 40×). (**C,D**) H&E stain reveals meningothelial cells featuring whorls, frequent intranuclear pseudo-inclusions (blue arrows), and some nuclear holes (40×). H&E, hematoxylin and eosin stain.

Histologic examination (using hematoxylin and eosin) revealed a meningothelial neoplasm with whorls, interlacing bundles of meningothelial cells, and extensive psammomatous calcifications (Figures 4A,B).

The intervening meningothelial cells display frequent intranuclear pseudo-inclusions and some nuclear holes (Figures 4B,C). There is no evidence of atypical features, including increased mitotic figures, hypercellularity, prominent nuclei, areas of small cell changes with high nuclear/cytoplasmic ratio, sheeting, and foci of spontaneous necrosis. There is no normal spinal parenchymal tissue, and no tumor invasion is identified.

Immunohistochemical stains with an appropriate control are performed, showing diffuse reactivity for epithelial membrane antigen (EMA) (Supplementary Figure S1A) and progesterone receptor (Supplementary Figure S1B). The proliferative index using Ki-67 is low and estimated at 2%–3% (Supplementary Figure S1C).

The overall pathologic findings are that of psammomatous meningioma, CNS WHO grade 1.

#### 2.3.2. Hospital course

After the surgery, the patient was started on physiotherapy and showed mild improvement in caudal anesthesia. During the hospital stay, the patient maintained her level of consciousness, oral diet was tolerated, and she had good bowel motion. She is currently being followed up in the clinic [out patient department, (OPD)] every 3 months.

## 3. Discussion

Spinal meningiomas are usually benign lesions and are mostly intradural extramedullary lesions. In rare situations, they can also be extradural tumors. The incidence of intradural extramedullary meningioma with extradural extension, as in the presented case, remains unknown.

Spinal dumbbell-shaped meningioma is a rare condition and is usually mistaken preoperatively for schwannoma, with only 21 dumbbell-shaped spinal meningiomas being reported from 1997 to 2017 (14).

In a study of 54 patients from 1963 to 1994, 47 tumors were intradural, 5 were epidural, and 2 were epidural and intradural (15). In this study, the rarity of this incidence is noted.

The concept of why intradural meningiomas could have an extradural extension is not well established but is explained in the hypotheses Zevgaridis and Thomé (3).

(I) By proliferation of ectopic arachnoidal cells around the periradicular nerve root sleeves;
(II) By the displacement of the primitive embryonic remnants of the arachnoid mater and villi along the periradicular dura; and
(III) By the migration of islands of arachnoid tissue into the extradural space.

In the presented case and during the initial assessment of the patient by a CT scan for the whole spine followed by a whole spine MRI, the lesion was highly suspected to be spinal schwannoma due to the presence of intradural and extradural components extending to the foramen. The patient was examined and assessed to rule out the presence of neurofibromatosis. Depending on our patient presentation, we indicate that spinal meningioma can be rapidly progressive despite being a lower-grade tumor. The course of the symptoms and the duration of deterioration were estimated to be 3 months. Despite visiting multiple health facilities, the fact that the patient had progressive weakness and neurological deficit and that the diagnosis was missed indicates that spinal lesions can be easily missed due to the suspension of muscular pain or if the patient had a history of spinal disc disease.

## 4. Conclusion

We recommend maximizing the decompression during surgery to ensure enough decompression and maximize the surgical benefits. Complete resection is recommended considering that excessive manipulation of the spine can produce fatal outcomes. Psychological support is an essential part of the treatment, and multidisciplinary team involvement is recommended; in particular, the involvement of a rehabilitation physician, physiotherapist, psychiatrist, and in our case, OBGYN, was necessary.

Meningiomas can be easily missed in diagnosis depending on imaging and the pathognomonic pattern it represents, which can mimic other pathologies, such as schwannomas. Hence, surgeons should always suspect their patient having a meningioma even if the pattern is not typical. Preoperative preparation must be taken, such as navigation and defect closure, in case it turned out be a meningioma instead of the presumed pathology.

### 4.1. Patient perspective

At first, I was very terrified and skeptical due to my symptoms, believing that there was something serious going on with all that stress and me going through pregnancy. I was a mess. When I turned up to FA and his team at King Fahad University Hospital, they assured me that my symptoms were due to a condition involving my spine and that it was treatable surgically. My symptoms improved, and all my fears faded away, thanks to this amazing team. I consent and understand that the information regarding my condition will be used for research purposes.

## Data Availability

The original contributions presented in the study are included in the article/**Supplementary Material**, further inquiries can be directed to the corresponding author.
