# Supplementary figures and images for: Intradural extramedullary spinal cord meningioma with a rare extradural foraminal extension: A case report
Source: Front Surg. 2023 Apr 17;10:1077355. doi: 10.3389/fsurg.2023.1077355 (PMC10150021; doi:10.3389/fsurg.2023.1077355)

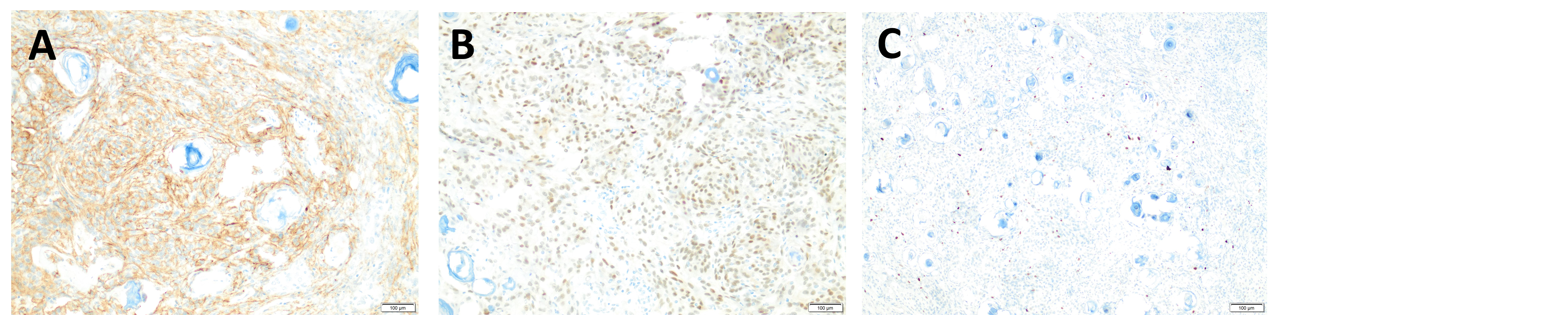

Supplement: Supplementary file 1 [file Image1.tiff]
